# Synthesis and Characterization of Cellulose and IPN (Cellulose/PVA) Hydrogels and Their Application in Dye Retention

**DOI:** 10.3390/polym18030346

**Published:** 2026-01-28

**Authors:** Meriem Mihoub, Salah Hamri, Marcel Popa, Camelia Elena Tincu (Iurciuc), Tewfik Bouchaour, Lamia Bedjaoui-Alachaher, Usman Abubakar Katsina, Mutawakkil Muhammad

**Affiliations:** 1 Laboratory of Research on Macromolecules (LRM), Faculty of Sciences, Department of Chemistry, University Abou Bakr Belkaid, BP119, Tlemcen 13000, Tlemcen, Algeria; meriem.07m@hotmail.fr (M.M.); salah_hamri@yahoo.fr (S.H.); bouchaour@yahoo.fr (T.B.); l_bedjaoui@yahoo.fr (L.B.-A.); 2 Centre de Recherche Scientifique et Technique en Analyses Physico-Chimiques (CRAPC), BP384, Bou-Ismail 42004, Tipaza, Algeria; 3 Faculty of Chemical Engineering and Environmental Protection “Cristofor Simionescu”, Department of Natural and Synthetic Polymers, “Gheorghe Asachi” Technical University, Bd. D. Mangeron, nr. 73, 700050 Iasi, Romania; 4 Academy of Romanian Scientists, Ilfov Str., nr. 3, Sector 5, 050045 Bucharest, Romania; 5 “Ioan Haulica” Research Institute, Faculty of Medicine, “Apollonia” University of Iasi, 700511 Iasi, Romania; camelia_tincu83@yahoo.com; 6 Department of Pure and Industrial Chemistry, Bayero University, Kano PMB 3011, Nigeria; aukatsina.chm@buk.edu.ng; 7 Department of Science and Technology Education, Bayero University, Kano PMB 3011, Nigeria

**Keywords:** cellulose, poly(vinyl alcohol), Tubantin Blue (DB78), dye adsorption, IPN hydrogels, epichlorohydrin

## Abstract

The discharge of dye-contaminated effluents from textile industries into water bodies poses a severe threat to aquatic ecosystems and human health. To address this challenge, cellulose and interpenetrating polymer network (IPN) hydrogels based on cellulose and poly(vinyl alcohol) (PVA) were developed via an in situ synthesis method. The cellulose solution was obtained by cold dissolving the polysaccharide in NaOH, then dissolving PVA. The IPN hydrogels were obtained by co-cross-linking the two polymers in an alkaline medium using ECH. To optimize the hydrogels, synthesis parameters like time (4–7 h), temperature (50–80 °C), and cross-linking ratio (ECH = 50–125% *w*/*w*) were varied. Different hydrogel compositions (Cel/PVA = 90/10 to 60/40 *w*/*w*) were tested for their absorption efficiency in removing Tubantin Blue (DB 78) dye under varying initial concentrations and temperatures. Hydrogels exhibit varying adsorption capacities for DB78, depending on their IPN composition, synthesis parameters, and dye concentration. Specifically, IPN adsorption capacity ranges from 8.8 to 38.1 mg DB78/g hydrogel (7.5–36.2% efficiency). At high effluent concentrations, IPN can reach a retention capacity of 217.7 mg/g, achieving a retention efficiency of 58.4%. Cellulose and cellulose/PVA IPN hydrogels show promise as sustainable adsorbents for treating dye-contaminated wastewater.

## 1. Introduction

Dye pollution from industrial effluents has become a critical environmental concern due to the extensive use of synthetic dyes in sectors such as textiles, leather, and paper manufacturing [1,2]. These dyes are often chemically stable, non-biodegradable, and toxic, posing significant threats to aquatic ecosystems and human health [3,4]. The presence of dyes in industrial wastewaters reduces light penetration [5], disrupts photosynthetic processes [6], and introduces carcinogenic and mutagenic compounds [7,8], thereby endangering biodiversity and public safety. Conventional treatment methods often fail to effectively remove these pollutants, necessitating the development of innovative and sustainable solutions. Among these, materials such as hydrogels (three-dimensional networks of polymers) and semi- and full-interpenetrated polymer networks (IPNs) have attracted attention for their ability to adsorb dyes efficiently [9,10]. These materials, particularly those derived from renewable sources such as cellulose, offer eco-friendly, reusable, and highly effective alternatives for dye retention and removal, thereby contributing to sustainable water management practices [11,12]. Cellulose-based hydrogels are polymeric networks that can absorb and retain large amounts of water or biological fluids without dissolving [13,14]. They have recently been explored due to their hydrophilic nature, attributed to their abundant hydroxyl groups, which enable strong water interactions, and to their cross-linking, which enhances structural stability. Their unique swelling properties [15], biocompatibility [16], biodegradability [17], and tunable mechanical characteristics [18] make them promising materials for various applications, including drug delivery [19], tissue engineering [20], wound healing [21], and environmental remediation [22].

Similarly, interpenetrating polymer networks (IPNs) are advanced materials formed by combining two or more polymers in a cross-linked structure. Networks can be of the semi-IPN type, when the linear chains of one polymer cross the meshes of the three-dimensional network of another polymer, or of the full-IPN type when both polymers form cross-linked structures that interpenetrate (either through simultaneous or consecutive cross-linking), or one linear polymer is cross-linked in the presence of another polymer-based cross-linked structure [23,24]. This unique structure imparts IPNs with enhanced mechanical strength, chemical stability, and improved swelling properties compared to single-polymer systems [25,26]. In recent years, IPNs have gained significant attention in water treatment applications due to their ability to efficiently adsorb pollutants, including organic dyes from aqueous solutions [27,28]. By combining cellulose, a renewable and biodegradable polysaccharide, with poly(vinyl alcohol) (PVA), a synthetic polymer known for its flexibility and hydrophilic nature, cellulose/PVA-based IPNs offer a synergistic approach to developing sustainable, high-performance materials for dye retention and wastewater treatment [29,30]. Moreover, hydrogels and IPNs, such as cellulose/PVA type, are emerging as promising materials for dye retention due to their exceptional adsorption efficiency, environmental compatibility, and reusability [31,32]. The inherent porous structure of cellulose-based hydrogels provides a high surface area and efficient interaction with dye molecules, thereby enhancing retention capacity [33]. Their renewable and biodegradable nature aligns with the growing demand for environmentally friendly solutions to mitigate pollution. Furthermore, incorporating PVA into IPNs enhances mechanical strength and stability while preserving biocompatibility, enabling repeated use without significant performance loss [34,35]. These attributes make cellulose-based hydrogels and IPNs sustainable and cost-effective candidates for wastewater treatment applications.

This study focuses on synthesizing and characterizing cellulose-based hydrogels and their IPNs with PVA to develop materials with improved synergistic features for dye-absorption applications. Cellulose, with its abundant hydroxyl groups, forms strong hydrogen bonds, making it a promising candidate for hydrogel synthesis. IPNs, formed by combining two or more polymers without creating a copolymer, offer synergistic properties, including enhanced mechanical stability, greater swelling capacity, and improved dye adsorption efficiency. The research seeks to optimize the synthesis conditions of cellulose hydrogels and cellulose/PVA (Cel/PVA) IPNs, investigate their structural and morphological attributes, and evaluate their functionality in dye retention. The hydrogels and IPNs were prepared using epichlorohydrin (ECH) as the cross-linking agent, while key synthesis parameters, including temperature, cross-linking duration, cellulose concentration, and polymer/ECH ratio, were varied to study their effects on the materials’ physical properties, particularly swelling behavior. The cellulose-based hydrogels and IPNs obtained were evaluated for dye adsorption, specifically targeting Tubantin Blue (DB78).

DB78 is a water-soluble anionic direct dye, typical for dyeing cotton fibers/fabrics and, therefore, cellulose. Its retention by cellulose is based on numerous physical interactions (Van der Waals forces and hydrogen bonds), which are favored by the presence of hydroxyl groups. It has an aromatic polynuclear structure with –SO_3_^−^ Na^+^ type substituents, which confer anionic character and high water solubility. It achieves color through physical interaction with the fiber rather than chemical bonding; it can be used at low temperatures in the dyeing process (e.g., 60 °C or 75 °C), thereby increasing its use and reducing energy consumption. The DB78 can be dangerous to human life and vegetables; it can effectively reduce the sunlight that reaches different vegetables, disrupting photosynthesis and affecting the ecosystem [36]. It can also cause eutrophication, leading to algal blooms and oxygen depletion. High doses of certain dyes can induce fatal serotonin toxicity in humans [37]. This dye can accumulate in the tissues of aquatic organisms and release carcinogenic compounds, posing a serious health risk to humans when consumed through the food chain. Its high chemical stability, determined by its aromatic structure, makes it persistent in aqueous environments and difficult to remove.

Hydrogels are materials known for their high capacity to retain various pollutants from the aqueous environment. Their retention in the material is based on a more complex mechanism than simple mechanical absorption in the hydrogel’s pores (sorption in the hydrogel matrix). In the case of DB78 (and other water-soluble dyes) the retention mechanism is a combination of its diffusion process in the polymer network, facilitated by the penetration by absorption of water that also carries the dye, and the process of its retention at the active sites of the polymer, which is an adsorption process based on the manifestation of physical processes and weak interactions (hydrogen bonds, Van der Waals forces). So, even if in the first stages we speak of an absorption process, because the dye is finally fixed by physical interactions (except in cases where it chemically binds to the polymer chains), the entire dye retention mechanism we will call it adsorption, and the material that retains it will be called an adsorbent.

The reason for undertaking this research is therefore determined, on the one hand, by the need to remove from wastewater this highly polluting dye widely used in the textile industry, and, on the other hand, by the material capable of retaining it to a large extent: a cellulose-based hydrogel, which has a high affinity for it. The dye’s characteristics make it a representative and relevant tool for evaluating the efficiency of new adsorption materials in treating colored wastewater.

Another argument for our study is that studies on the adsorption of this dye from textile industry wastewater are scarce in the literature. Various chemically adsorbent materials, including irregular particles, micro- and nanoparticles, and hydrogels, have been used for this purpose. Irregular eggshell particles with a surface area of 1–1.5 cm^2^ are effective adsorbents of DB78 [38]. Coal combustion ash, which is a pollutant, has been modified by a thermochemical process to increase its specific surface area and used with good results to retain DB78 from wastewater [39]. Another research group prepared nickel ferrite- and hazelnut-shell-based activated carbon nanocomposites via the hygrothermal method and used them as adsorbents for removing anionic direct dyes (Direct Red 31 (DR31) and DB78) from wastewater. The retention mechanism involves physiosorption, during which electrostatic adsorption is the primary driving force [40]. Adsorbent materials based on polysaccharides are also reported in the literature. Bassyouni et al. report the capacity of chitosan- and nanocellulose-based microbeads to retain DB78 as a function of the adsorbent concentration, contact time with the dye, its initial concentration, and the weight of nanocellulose in the microbeads. Dye retention is based on the adsorption of the dye onto the cellulosic material’s surface [41]. Chitosan-based microbeads, this time incorporated into a poly(acrylamide) gel, are used by the same author to retain direct dyes (including DB78) from wastewater [42]. Murcia-Salvador et al. used spherical chitosan particles and a cyclodextrin-epichlorohydrin polymer as adsorbents and studied the adsorption of DB78 using three adsorption isotherms [43]. Cellulose-based hydrogels used as adsorbents for DB78 are not reported in the literature. One can mention, for example, only one type of hydrogel, namely chitosan-based spherical particles and nanocrystalline cellulose obtained from Coconut husk fiber, used to retain cationic dyes from wastewater [44,45].

In this work, two types of adsorbents were synthesized: one based on cellulose and the other on a full-IPN of cellulose and poly(vinyl alcohol) (Cel/PVA). The adsorption efficiency of DB78 under various conditions, including different temperatures, dye concentrations, and IPN compositions, was investigated.

The results showed that the Cel/PVA IPNs exhibited enhanced swelling and DB78 adsorption. These findings highlight their potential as effective and sustainable materials for wastewater treatment. This aspect of the study is particularly relevant for environmental applications, as the removal of dyes from wastewater is a critical challenge in the textile and dyeing industries.

## 2. Materials and Methods

### 2.1. Materials

The materials used in this study include α-cellulose biopolymer powder, epichlorohydrin (ECH) as a cross-linking agent, poly(vinyl alcohol) (PVA) polymer powder (molecular weight 89.000–98.000, 99% hydrolyzed), and sodium hydroxide (NaOH). All chemicals were purchased from Sigma-Aldrich (Merck KGaA, Darmstadt, Germany) and used without further purification. Tubantin Blue (DB78) (Sigma-Aldrich, Merck KGaA, Darmstadt, Germany) was selected as the model dye to evaluate the adsorption performance of the synthesized cellulose-based materials.

### 2.2. Cellulose Hydrogel Synthesis

The cross-linking of cellulose was performed at a basic pH, using a 3% (*w*/*v*) cellulose solution dissolved in aqueous sodium hydroxide (NaOH) at low temperature (T = −60 °C). The dissolution process of cellulose powder in the NaOH solvent is carried out in two steps to ensure a homogeneous solution. In the first step, a 6% (*w*/*v*) NaOH solution (50 g) is cooled to −6 °C, and powdered cellulose is gradually added while stirring at 11,000 RPM for 3 min. In the second step, a 9% (*w*/*v*) NaOH solution (25 g) is added to the mixture, and stirring is continued for an additional 3 min. This process results in a semi-transparent, homogeneous cellulose solution, which is stored at 5 °C for further use. A 42 g sample of the cellulose solution was weighed. The corresponding volume of epichlorohydrin (ECH) was calculated using its density (v = m/ρ, ρ = 1.18 g·mL^−1^). The calculated volume of ECH was added dropwise to the cellulose solution using a micropipette, and the mixture was stirred for 5–10 min. Subsequently, the mixture was placed in an ultrasonic bath for 10 min to eliminate air bubbles formed during stirring. The resulting solution was then poured into a mould (beaker) while being weighed and subsequently placed in an oven set at 60 °C.

The cross-linking agent, ECH, was then added and mixed under intense stirring. The reaction mixture was heated to a high temperature for a certain period of time in cylindrical glass beakers for cross-linking. The obtained hydrogels were thoroughly washed with distilled water to remove any unreacted components and then dried. In this study, the synthesis parameters of cellulose hydrogels (HC) were varied, including the cross-linking agent (ECH/cellulose, % *w*/*w*) ratio, cross-linking temperature, and reaction duration, to evaluate their influence on hydrogel properties. The hydrogels with different parameters are synthesized following the experimental plan presented in Table 1. For each parameter that varied (ECH%, temperature, duration), the rest of the parameters are kept constant:

### 2.3. Synthesis of the Cel/PVA IPNs

The full-IPNs (Cel/PVA) were prepared by in situ cross-linking. This method is feasible because both PVA and cellulose can be dissolved in the same solvent (water) and cross-linked simultaneously using ECH as the cross-linking agent [46,47]. The dissolution process is carried out in two steps to ensure a homogeneous solution. In the first step, a 6% (*w*/*w*) NaOH solution (50 g) is cooled to −6 °C, and powdered cellulose is gradually added while stirring at 11,000 RPM for 3 min. In the second step, a 9% (*w*/*w*) NaOH (25 g) solution is added to the mixture, and stirring is continued for an additional 3 min. This process results in a semi-transparent, homogeneous cellulose solution, which is then stored at 5 °C for further use. A powdered PVA was then added in small quantities to the cellulose solution to obtain the following compositions (cellulose/PVA, *w*/*w*): (90/10, 75/25, 60/40, and 50/50), while stirring at high speed for 8 min until a homogeneous mixture is obtained. After that, 75% of ECH (*w*/*w*, based on the cellulose solution) is added to the cellulose-PVA solution; the ECH cross-linker is introduced dropwise under continuous stirring for 8 min to ensure uniform dissolution. The resulting mixture is then transferred into small cylindrical molds (a beaker with a 25 mL capacity and a 3.2 cm diameter) and cured at 60 °C for 5 h. To assess the influence of temperature on hydrogel properties, a sample was also synthesized at 50 °C, with the other reaction conditions held constant. After curing, the IPN samples are thoroughly rinsed with distilled water to remove residual NaOH, unreacted polymers, ECH, and other impurities. The samples are air-dried for 24 h, followed by vacuum drying to ensure complete water removal. IPNs with different ratios (Cel/PVA) as cited above were obtained and stored for characterization. These compositions allow the investigation of the effects of polymer ratios on the properties of the IPNs.

**Figure 1:**
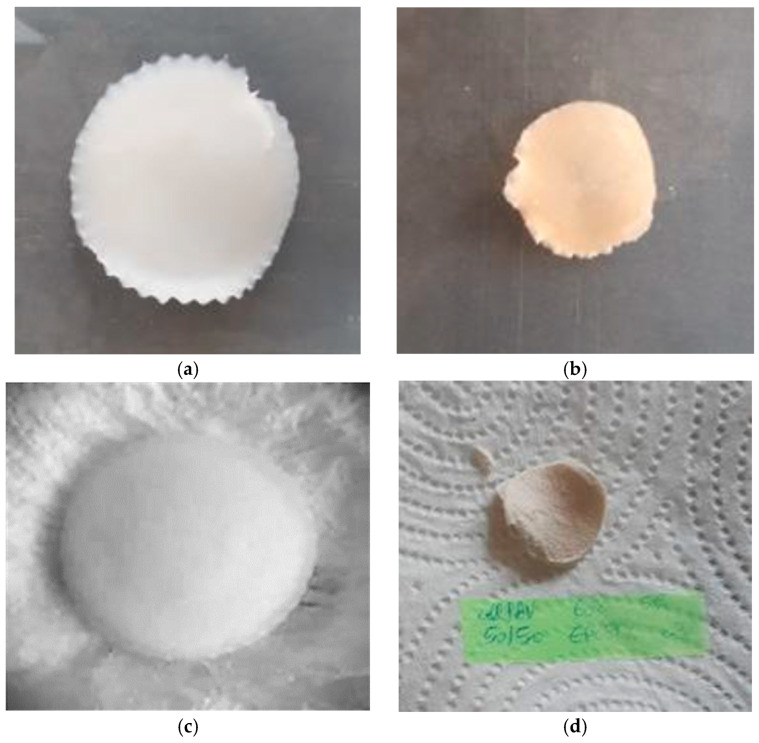
Images of cellulose hydrogel (HC) and Cel/PVA IPN (50/50 *w*/*w*): (**a**)—HC washed after synthesis, (**b**)—HC dried, (**c**)—full-IPN Cel/PVA synthesized by the in situ method and washed, (**d**)—IPN Cel/PVA after drying.

Figure 1 shows photographs of a cellulose hydrogel (HC) and an IPN hydrogel (50/50 *w*/*w* Cel/PVA) before and after drying.

### 2.4. Materials Characterization

Structural characterization of the as-synthesized materials was performed using **Fourier-transform infrared (FTIR)** spectroscopy, and morphological characterization was performed by scanning electron microscopy (SEM). A PerkinElmer 2000 FTIR model (PerkinElmer, Inc., Shelton, CT, USA) was used to confirm the chemical structures of samples on a NaCl plate. The FTIR spectra of all the samples were recorded over the 4000–400 cm^−1^ range, with a resolution of 4 cm^−1^, and 16 scans at 20 °C. **SEM micrographs** of the samples were obtained using a scanning electron microscope (Hitachi SU-1510, Hitachi Company, Chiyoda City, Tokyo, Japan) to examine their surface morphology and structural features.

### 2.5. Swelling Measurements

The swelling characteristics of hydrogels in aqueous environments are essential for their intended use, as the dye’s diffusion into the mesh of the network depends mainly on them. A kinetic study of swelling in distilled water (neutral pH) at room temperature (20 °C) was performed to compare the influence of synthesis parameters on the swelling rate of hydrogels prepared under different conditions.

Pre-weighed, dried hydrogel samples (the cellulose hydrogel samples weighed between 160–240 mg, while the Cel/PVA IPNs samples ranged from 64–115 mg) were immersed in 25 mL aqueous media (distilled water, pH = 7) at 20 °C. At precise time intervals, hydrogels were weighed after removing excess waterfrom the sample’s surface with superabsorbent tissue paper, until there was no change in hydrogel weight [48,49]. The swelling ratio (Q, %)was calculated using Equation (1), as follows:

(1) $$Q\%=\frac{W_{s}-W_{d}}{W_{d}}\times 100$$

where *W_s_* and *W_d_* are the weights of swollen and dried hydrogel, respectively. Swelling measurements were repeated three times to confirm the results.

The degree of swelling was measured three times, and the results are reported as the average ± STDEV.

The normalized degree of swelling (α) is the degree of swelling at time t (Qt) divided by the degree of swelling at equilibrium (Q_eq_) [50].

(2) $$\alpha =\frac{Q_{t}}{Q_{eq}}$$

Swelling degree kinetics

The swelling kinetics study was conducted to analyze the swelling results. Different mathematical models were used to calculate the swelling kinetics constants. The swelling kinetics are best explained by the mathematical model that exhibits the highest correlation coefficient (R^2^). In this study, the mathematical models used to analyze the swelling kinetics were:

The model by Peppas [51] can be represented as

(3) $$\alpha ={k\times t}^{n}$$

α represents the normalized degree of swelling. The exponential factor *n* describes the type of transport mechanism. The hydrogel constant is denoted by k, and t represents the swelling time.

The 1st order kinetics can be expressed as follows:

(4) $$\alpha =\left(1-{A\times e}^{-kt}\right)$$

where A is the pre-exponential factor.

The second-order kinetics can be expressed as follows.

(5) tMt=1k×M∞2+(1M∞)×t

The mass of the hydrogel at equilibrium is denoted as M∞, while Mt represents the mass of the hydrogel at time t [52]. The swelling kinetics of the hydrogels were investigated to fit the swelling behavior data to the modified Voight equation, where β is not equal to 1.

(6) $$\alpha =1-e^{{-(t/\tau )}^{\beta }}$$

where τ is a parameter that represents the equilibrium swelling rate and can be found by graphing ln[−ln(1 − α)] against ln(t), β is the β = slope, and lnτ = −Intercept/β, resulting in τ = $e^{\left(-\;\frac{intercept}{\beta }\right)}$.

### 2.6. Determination of the Capacity of Hydrogels to Retain DB78 from Aqueous Solutions

The conditions for establishing and plotting the calibration curve:

For a 0.1 mg/mL aqueous solution of DB78, the maximum absorption wavelength was determined by UV–Vis spectrophotometry at λ = 600 nm.

**Figure 2:**
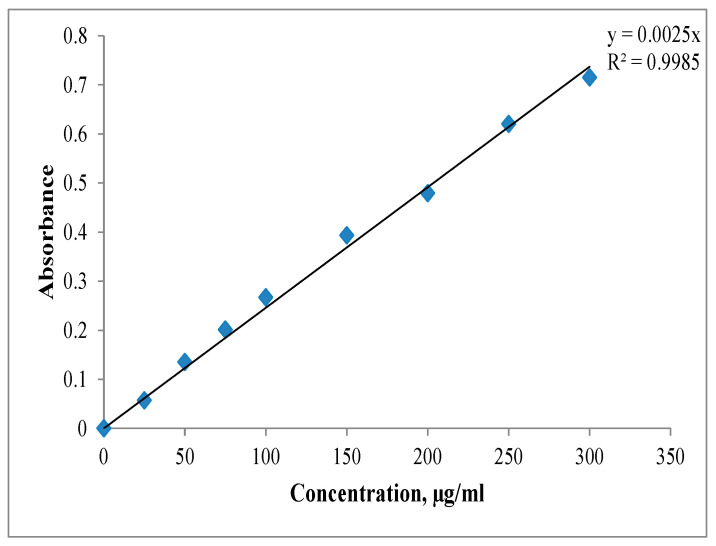
Calibration curve for DB78 dye.

A standard dye solution was prepared (c = 5 mg/mL), from which, by dilution, several solutions (c = 25–300 µg/mL) were obtained. The absorbance of these solutions was measured using a UV-Visible spectrophotometer (a double-beam Varian Cary 100, Varian, Inc., Mulgrave, Vic, Australia). The results allow us to draw the calibration curve shown in Figure 2.

To examine the dye retention capability of the IPNs, colored aqueous solutions of DB78 were prepared, and the HC and IPN samples were immersed in them; adsorption kinetics were monitored using a double-beam UV-Visible spectrophotometer (Varian Cary 100, Varian, Inc., Mulgrave, Vic, Australia).

We studied the absorption kinetics of DB78 using HC and IPN hydrogels synthesized with varying cross-linking agent concentrations, at different times and temperatures, and at different DB78 solution concentrations, across different Cel/PVA ratios. To study the adsorption kinetics of DB78 on hydrogels obtained with different EPC concentrations, at different times and temperatures, and with different Cel/PVA ratios, the sample to be analyzed was immersed in vials containing 5 mL of a 5 mg/mL DB78 solution. During the experiment, the flasks were sealed to prevent water evaporation. The experiment was performed at 25 °C. At different time intervals, 100 µL of solution was taken and diluted to 10 mL in a 10 mL volumetric flask. The concentration of the resulting solution was determined using a UV-Vis spectrophotometer at a wavelength of 604 nm. A volume of 100 µL of water was reintroduced into the flask to maintain the solution’s total volume, and the necessary correction was applied when performing the calculations. The operation is repeated until adsorption equilibrium is reached (after 48–96 h, defined as the contact time).

To study the adsorption kinetics of DB78 at different concentrations in aqueous solution, 5 mL of solutions containing 2.5, 5, 7.5, and 10 mg/mL were prepared. Accurately weighed amounts of hydrogel were added to the solution vials. At different time intervals, 100 µL of solution was taken, diluted to 10 mL with distilled water, and the absorbance was determined. The subsequent procedure was identical to the one previously described for studying the influence of the other parameters.

All experiments were performed in triplicate, and the reported values are expressed as mean ± standard deviation.

## 3. Results and Discussion

**Figure 3:**
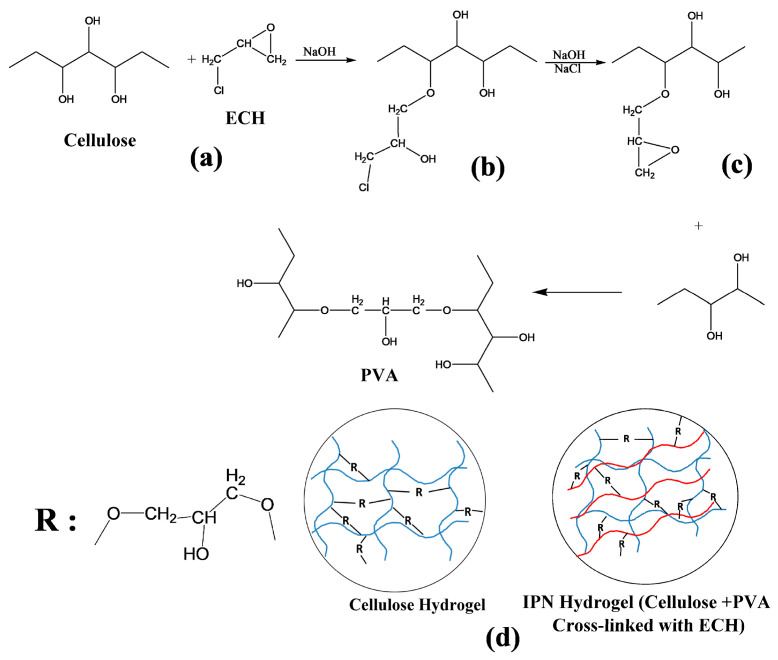
Schematic presentation of the cross-linking reaction of polymers with ECH, and (**a**–**c**) represent the stages of the cross-linking reaction; (**d**) represents the schematic structures of the cellulose-based hydrogel, respectively, of the IPN type-the red line represents the poly(vinyl alcohol)-PVA and R represents the radical from the epichlorohydrin linkage, which forms the bridge between two polymer chains.

The preparation of cellulose hydrogels (HC) and an IPN based on cellulose and PVA is based on a cross-linking reaction promoted by epichlorohydrin in a strongly alkaline medium. The reaction proceeds in three stages, shown schematically in Figure 3: the first consists of the opening of the epoxide ring of ECH by the addition of the –OH group from cellulose or PVA (stage a), followed by the restoration of the epoxide ring activated by NaOH (stage b) and finally the addition to the newly formed epoxide ring of another hydroxyl group (from the cellulose or PVA chain) (stage c). In this last stage, the transverse bridge between two polymer chains forms, ensuring cross-linking. As suggested by Figure 3d, the rest of the epichlorohydrin that ensures cross-linking can be achieved either between two polymer chains from the same polymer, or coming from the two polymers in the mixture. As a result, the network formed is very complex, of the interpenetrating/interconnected type.

### 3.1. FTIR Spectroscopy of Cellulose Hydrogels and IPN

**Figure 4:**
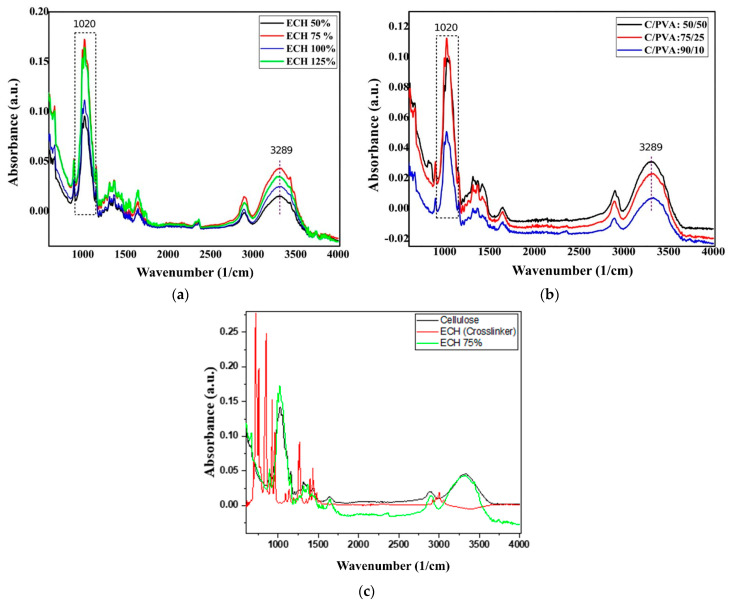
(**a**) FTIR spectra of cellulose-based hydrogels (HC) prepared with different ECH cross-linking ratios; (**b**) FTIR spectra of cellulose–PVA interpenetrating polymer network (IPN) hydrogels with different compositions; (**c**) FTIR spectra comparing native cellulose, the ECH cross-linker, and the cellulose hydrogel cross-linked with 75% ECH.

Figure 4a shows the FTIR spectra, which provide information on the chemical structure of cellulose hydrogels synthesized with different ECH cross-linking ratios (at 60 °C for 5 h, with a 3% cellulose (*w*/*w*) concentration).

The absorbance peaks at 3289 cm^−1^ and 1020 cm^−1^ in Figure 4a were assigned to O–H and C–O vibrations. The C–O bond formation around 1020 cm^−1^ could be attributed to the reactions between the ECH molecule and the cellulose, while the broad peak at 3289 cm^−1^ for the O–H group involves vibrations of intra-molecular and intermolecular hydrogen bonds in cellulose [53,54]. Notable changes include an increase in the C–O stretching intensity at 1020 cm^−1^, indicating successful cross-linking, and the O–H stretching band around 3289 cm^−1^, which becomes more pronounced with higher ECH content. These shifts suggest an increase in the formation of new ether bonds and hydrogen bonds as the ECH ratio increases, confirming the progressive cross-linking between cellulose chains [55]. The O–H stretching band at 3289 cm^−1^ also becomes more pronounced, indicating additional hydrogen bonding due to the hydroxyl groups introduced by the ECH.

At an intermediate ECH content of 75%, cross-linking is probably optimal, leading to a more efficient polymer matrix and an increase in the number of hydrogen bonds, reflected in a higher-intensity absorption peak. At higher ECH concentrations (100%), the increased cross-linking density may restrict polymer chain mobility and reduce the number of free hydroxyl groups available for hydrogen bonding, thereby decreasing the apparent intensity of the absorption peak. At 125% ECH, excess cross-linking agent may lead to the formation of additional ether bonds and the presence of unreacted functional groups, which can cause a further increase in the absorption peak intensity [56,57]. Figure 4b represents FTIR spectral comparison between the different compositions of IPN (Cel/PVA): (50/50), (75/25), and (90/10). The spectra show the main cellulose and PVA signals: a broad O–H band (~3200–3500 cm^−1^), C–H stretching (~2900 cm^−1^), and C–O/C–O–C stretching (~1000–1120 cm^−1^). As cellulose content increases, the O–H band broadens and shifts slightly, indicating stronger hydrogen bonding. In contrast, PVA-rich samples show higher intensity in the ~1080–1100 cm^−1^ region, due to PVA’s strong C–O contributions. Overall, cellulose-rich IPNs form stronger hydrogen-bonded networks, while PVA-rich IPNs display more free –OH groups and enhanced C–O stretching, which explains their different swelling and dye adsorption behaviors. Figure 4b demonstrates a reduction in the band intensity at 1020 cm^−1^ as the cellulose: PVA ratio shifts from 75:25 to 50:50. This phenomenon can be attributed to the decrease in cellulose content within the polymer matrix. The band primarily corresponds to C–O vibrations in cellulose; thus, its intensity is expected to decrease as the cellulose proportion decreases, even when PVA is present. Furthermore, hydrogen bonding interactions between cellulose and PVA may influence band intensities without necessarily inducing significant shifts in the absorption bands.

An additional FTIR figure (Figure 4c) was included for comparing the FTIR spectra of native cellulose, the cross-linking agent ECH, and the cross-linked cellulose hydrogel with 75% ECH.

The lack of notable shifts in the absorption bands in the cellulose-based hydrogel spectrum suggests that the cellulose’s fundamental chemical structure remains intact. Cross-linking with ECH does not introduce new primary functional groups but instead causes structural reorganizations and alters the bonding environment of existing groups [57]. It is important to note that changes in FTIR band intensities are primarily qualitative and cannot be used as direct or quantitative measures of cross-linking. They are affected by overlapping vibrations, chain orientation, and the accessibility of functional groups. Therefore, the band at approximately 1020 cm^−1^, depending on the cellulose: PVA ratio, mainly reflects changes in composition and intermolecular interactions rather than the efficiency of the cross-linking process, while FTIR spectra provide qualitative support for network formation. In contrast, the functional confirmation of cross-linking and network architecture is primarily supported by swelling, stability, and transport results presented in the following sections.

### 3.2. Scanning Electron Microscope (SEM) Analysis

**Figure 5:**
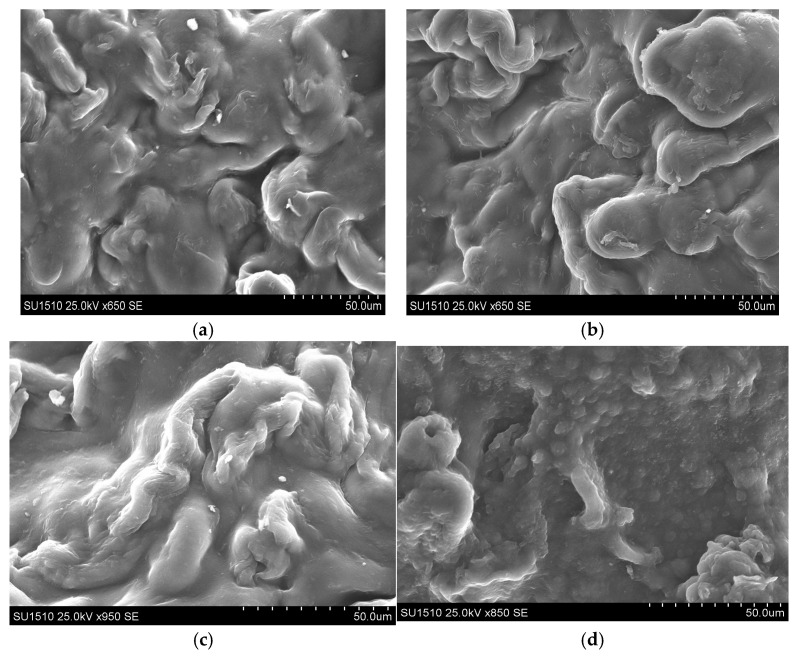
SEM photographs of cellulose hydrogels (with different amounts of ECH (50%—(**a**), 75%—(**b**), 125%—(**c**)) and IPN Cel/PVA ratio = 60/40 (*w*/*w*), 75% ECH—(**d**), (t = 5 h; T = 60 °C), (resolution 50 µm).

The SEM micro-images of cellulose hydrogels with varying ECH ratios (50%, 75%, and 125%) are displayed in Figure 5a–c below, which shows a non-porous, dense surface morphology [58]. The SEM images reveal that increasing the ECH ratio results in the formation of cellulose hydrogel with apparently denser, more compact morphologies. The network formed by hydrogen bonds between cellulose chains and the new –OH group introduced by ECH cross-linking contributes to the observed structure and swelling behavior [59]. The IPN exhibits a less compact morphology (Figure 5d) than the cellulose hydrogel obtained under the same conditions (Figure 5b), which is attributed to the incorporation of PVA, a less compact network that enhances swelling capacity and potentially improves dye absorption performance.

### 3.3. Swelling Study

**Figure 6:**
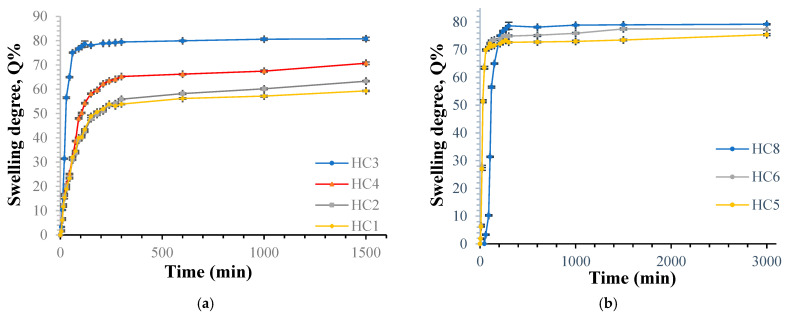

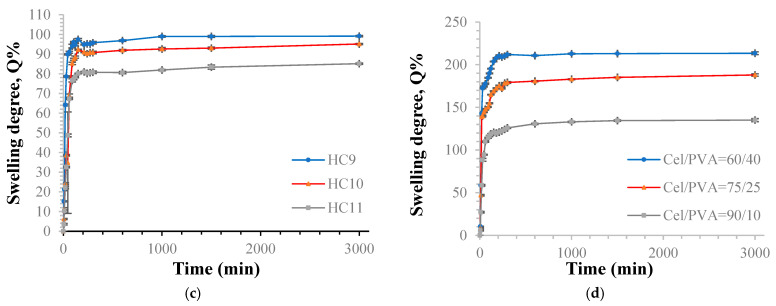
Swelling kinetics in distilled water of cellulose hydrogels (**a**) with different cross-linking agent ratios; (**b**) at different temperatures; (**c**) at different durations; and (**d**) Cel/PVA IPNs with different compositions.

Figure 6 shows the swelling measurements and their variations over time, illustrating how the ECH ratio, reaction temperature, and cross-linking duration affect the swelling of HC hydrogel in water. Also, Table S1 (Supplementary Information) lists the factors that affect the swelling degree.

Figure 6a shows the swelling behavior of cellulose hydrogels synthesized with varied ECH levels (50%, 75%, 100%, and 125%) (3% solution concentration of cellulose), cross-linked at 60 °C for 5 h. We observe that the swelling degree increases with increasing ECH, reaching a maximum of 75% swelling ratio, then decreases slightly to 67% at ECH 125%. These results exhibited an unusual trend, as observed in other studies [60,61], including the case of a hydrogel based on carboxymethyl cellulose cross-linked with epichlorohydrin, used as a support for the immobilization of furazolidone [62]. In principle, increasing the amount of cross-linking agent should increase the network density and decrease the mesh size. Consequently, the degree of swelling should decrease as the cross-linker concentration increases. A possible explanation for this unusual trend is as follows: swelling in water is influenced, on the one hand, by the network’s cross-link density (which decreases with this property) and, on the other hand, by the product’s hydrophilicity. If we consider the cross-linking mechanism, we see that each ECH molecule that creates a bridge between the cellulose chains is accompanied by the appearance of a new –OH group, which contributes to increasing the product’s hydrophilicity (see Figure 3). The final result of the competition between these two effects is, up to a certain cross-linker level, in favor of the second, so the degree of swelling can increase with the ECH level. At the same time, the introduction of the rest of the ECH between the polymer chains distances them and thus partially breaks the hydrogen bonds between the numerous hydroxyl groups, which contribute to the network density. As a result, the mesh size increases, allowing the diffusion of increasingly larger amounts of water. The greater the amount of ECH, the greater the number of hydrogen bonds broken, justifying the increase in the degree of swelling. Except for one particular case at ECH 125%, where the swelling rate does not follow this law; on the contrary, it decreases slightly below ECH 100%, but always remains above the ECH 50% and ECH 75% values. In this case, the network density probably becomes too high due to the participation of more and more hydroxyl groups in cross-linking, and the replacement of the network formed by these bonds with a stronger network (based on covalent bonds between polymer chains via ECH) and exceeds the effect induced by the increase in hydrophilicity and the attenuation of hydrogen bonds between –OH groups.

Figure 6b shows the swelling equilibrium of cellulose hydrogels (solution concentration of cellulose of 3%), cross-linked with 75% ECH, synthesized at different temperatures (50 °C, 60 °C, 80 °C), during a reaction time of 6 h. The curves showed a consistent rise initially, then stabilized after 4 h, with a swelling rate of 70–80%. From the results, we also observed that the swelling rate increases with increasing reaction temperature. But there are slight differences in the swelling rate values between the analyzed samples.

The temperature significantly influences chemical reactions; higher temperatures lead to faster and more vigorous reactions. In our case, the effect should be the intensification of the cross-linking reaction, so the consequence should be a decrease in the degree of swelling with increasing this parameter. The results are shown in Figure 6b; however, they are contrary to expectations: the swelling rate increases with increasing temperature! The plausible explanation in this case is that, by increasing this parameter, the viscosity of the reaction medium (which is initially high) decreases sharply with increasing temperature. The thermal agitation of the macromolecules increases, and even if the first step of the cross-linking mechanism, i.e., the reaction between the ECH and the –OH groups of the polymer chain intensifies, the second step, concerning the cross-linking itself, becomes increasingly less probable (this assumes the rapprochement of the cellulose chains to promote the formation of the cross-linking bridge—see the cross-linking mechanism, Figure 3).

Figure 6c shows the swelling equilibrium of cellulose hydrogels containing 3% (*w*/*v*) cellulose, cross-linked with 75% ECH, at a reaction temperature of 70 °C, synthesized at different durations (4, 5, and 7 h). The curves show a steady increase in swelling degree over the first 4–5 h, followed by an equilibrium range of 80% to 100%. The results also show that the swelling degree decreases with increasing reaction time, as expected, due to the progressive formation of a denser network.

The duration of the cross-linking process is a key factor. Extending it is expected to enhance cross-linking, resulting in structures with higher density and reduced water swelling. The results presented in Figure 6c confirm the theoretical considerations: the swelling kinetics are slower, and the maximum swelling degree reached after 3000 min decreases with increasing cross-linking duration.

Figure 6d reveals that the swelling kinetics of Cel/PVA IPNs with different compositions (90/10, 75/25, 60/40) synthesized with 3% (w%) of cellulose, at a temperature of 60 °C for 5 h duration, using 75% ECH, the maximum swelling rate is reached in half time (1600 min) compared to cellulose hydrogels, and reaching higher values 125% to 210%, and demonstrates that increasing the proportion of cellulose in the hydrogel composition results in a decrease in the swelling ratio. It was expected that increasing the cellulose content in the composition would increase the swelling ratio in water, given the polysaccharide’s higher hydrophilicity. However, the opposite effect was observed, as explained below. Even though cross-linking with ECH occurs at the –OH groups of cellulose and PVA, the probability of the cross-linker binding to the polysaccharide chains is higher, given, on the one hand, the higher number of –OH groups in the cellulose unit, and on the other hand, the greater distance of the primary –OH group (C6) of cellulose from the base chain, which means that steric hindrance is less evident than for PVA; for this, ECH has better accessibility to the –OH group, so the probability of the reaction is higher. Therefore, the swelling rate of hydrogels should increase with the decrease in the proportion of cellulose in their composition, which is, in fact, what happens with our hydrogels.

The addition of PVA also increases pore size, facilitating faster water uptake [63,64]. It should be noted that the swelling results for cellulose-based hydrogels are in good agreement with the electron microscopy images. It is evident that a more intense swelling kinetics, correlated with a higher maximum swelling degree, is observed for hydrogels containing PVA, which have a lower density. Table 2 shows the n, k, and τ values obtained by integrating the swelling data under different kinetic diffusion models.

Values of the exponential factor n in Peppas’ equation that are less than or equal to 0.5 indicate that the absorption mechanism of the aqueous solution is controlled by diffusion. Fickian diffusion occurs when the relaxation time (Tr) of the polymer is longer than the diffusion time (Td) of the solvent. Non-Fickian diffusion occurs when the relaxation time is roughly equal to the diffusion time, making Fick’s laws inadequate to explain it [65]. In the analyzed hydrogel films, diffusion generally follows Fickian behavior except for the samples with ECH concentrations of 100% or 125%. For the PVA-containing hydrogel films, we observe that the exponential factor n is small, approximately 0.1. According to the literature, the polymer’s high porosity may have led to a low n value in the Ritger-Peppas kinetic model, as the pores in the material allowed water to diffuse into the matrix almost instantly [66].

The data in Table 2 show that the hydrogel’s swelling behavior follows second-order kinetics, causing the swelling rate to decrease rapidly over time due to its dependence on changes in osmotic pressure. The value of τ indicates the swelling rate and reflects resistance to water diffusion. A lower τ value means a faster swelling rate [52,67]. From Table 2, we observe that as the maximum degree of swelling increases, the value of τ decreases.

It should be noted that the determinations of the degree of swelling (in triplicate) showed excellent reproducibility, with standard errors within the limits of ±0.1 and ±1.8, and are included in error bars in Figure 6.

### 3.4. DB78 Adsorption and Retention

The adsorption of DB78 in synthesized hydrogels is, of course, based on its diffusion into the network’s mesh, facilitated by the swelling of the network and the water that carries the dye. DB78 is a dye specific to cellulose, and its retention in hydrogel is influenced by its high hydrophilicity. Two factors are expected to contribute to DB78 retention: on the one hand, the network density (and the material’s porosity) and, on the other hand, its hydrophilicity.

**Figure 7:**
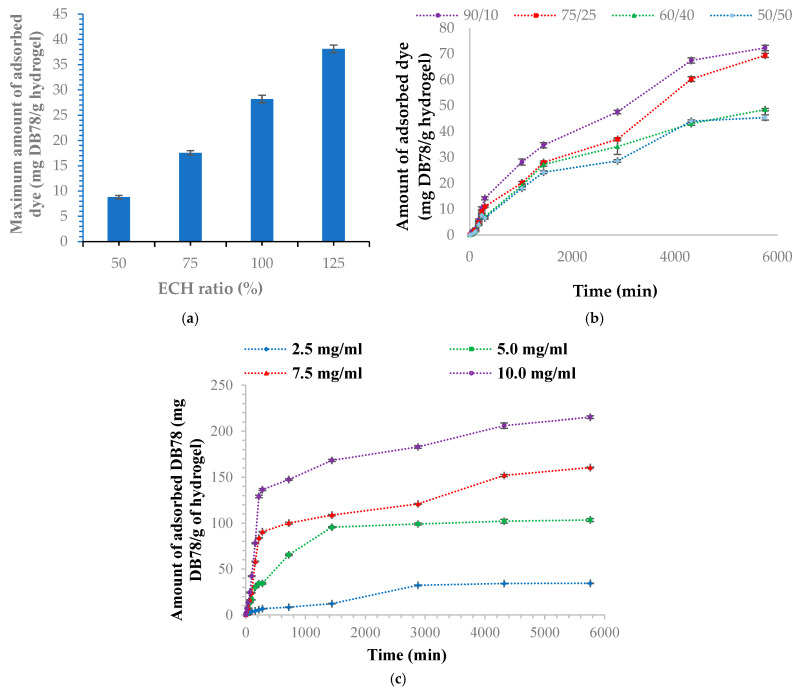
Adsorption kinetics of DB78 using (**a**) cellulose hydrogels with different ECH% (at equilibrium); (**b**) IPN at different Cel/PVA ratios; (**c**) Cel/PVA IPN (90:10) at different TBG dye concentrations. (**a**) shows the influence of the cross-linker concentration on the amount of dye adsorbed by the hydrogels at equilibrium. It should be noted that the hydrogels obtained with cross-linker concentrations of 50% and 75% adsorbed the dye faster, with equilibrium reached within 48 h. The maximum adsorbed dye, however, is reduced to 8.8 mg/g for the hydrogel cross-linked with 50% ECH and to 17.56 mg/g for the one cross-linked with 75% ECH. The hydrogels adsorb higher amounts of dye when cross-linked with higher ECH concentrations (100% and 125%), reaching equilibrium sorption of 28.2 and 38.1 mg/g, respectively. Still, the process is slower, and equilibrium is reached after approximately 96 h. In terms of adsorption efficiency, it ranges from 7.5% to 36.2% and increases with increasing cross-linker amount (Table S2).

Figure 7 shows the adsorption of DB78 dye by hydrogels with different ECH ratios (at equilibrium), the adsorption behavior of DB78 dye at different Cel/PVA IPN compositions, and the adsorption kinetics of DB78 dye at various concentrations.

It is important to note, however, that the results align well with swelling kinetic results, since the retention of the dye in the hydrogel is determined by diffusion, which in turn depends on hydrophilicity, especially on the cross-linking density of the network.

The inclusion of the second polymer (PVA) within the network increases the separation of cellulose chains, thereby expanding the network’s mesh size. Consequently, this modification enhances the network’s capacity to absorb a greater amount of dye, even at 10% PVA concentration. In principle, the quantity of dye adsorbed is influenced by two primary factors: first, the increased hydrophilicity of cellulose and the dye’s higher affinity for this polymer; second, the network’s porosity and pore size, which affect the dye’s diffusion. The equilibrium between these two effects dictates the extent of dye adsorption. In the case of hydrogels composed solely of cellulose, the determining factors for the amount of DB78 adsorbed are the network mesh size and the hydrogel’s porosity. Adding a small amount of PVA to the formulation significantly changes the hydrogel’s structure, making it much more porous (see Figure 5d) with larger network pore sizes. This modification results in the adsorption of greater quantities of dye, even in comparison to hydrogels composed solely of cellulose, notwithstanding the fact that the material’s hydrophilicity has slightly diminished (PVA exhibits less hydrophilicity than cellulose). Subsequently, when considering only the IPN, it is observed that a reduction in cellulose content results in progressively lower dye adsorption. This phenomenon is theoretically governed by the dye’s diminishing affinity for the correspondingly smaller amount of cellulose within the IPN structure, notwithstanding the fact that the network’s permeability to water and dye may increase with increasing PVA content, thereby enhancing swelling. The efficiency of DB78 retention by these hydrogels also increases, too, with the amount of cellulose in their composition, varying between 28.5% si 42% (Table S2, Supplementary Material)

Figure 7c shows that a higher concentration of the DB78 solution leads to greater dye adsorption, due to a higher concentration gradient between the solution and the Cel/PVA (90:10) interpenetrating polymer network. This gradient drives the diffusion of dye molecules into the hydrogel network. Also, the amount of DB78 dye adsorbed (217 mg/g) at a higher concentration (10 mg/mL) is bigger than the quantity adsorbed (34.7 mg/g) at the concentration of (2.5 mg/mL, which proves also that the IPN (90:10) has a bigger adsorption capacity. Similarly, the efficiency of DB78 retention varies with dye concentration, ranging from 42% to 56% (Table S2, Supplementary Material).

Even though a systematic study on the influence of temperature synthesis on the DB78 adsorption capacity of IPN-type hydrogels has not been carried out, the two experiments carried out for the hydrogel with the composition 60/40 (Cel/PVA) at temperatures of 50 °C and 60 °C, respectively, revealed, as expected, an increase in the adsorption capacity with this parameter. Thus, the maximum adsorption capacity for IPN obtained at a temperature of 50 °C is 121.5 mg/g (corresponding to a dye retention efficiency of 53%), while for IPN synthesized at a temperature of 60 °C, an adsorption capacity of 140.2 mg/g is recorded corresponding to a TBG retention efficiency of 58.4% (Table S2, Supplementary Material). The explanation is simple: only for cellulose-based hydrogels does increasing the temperature during hydrogel synthesis result in a slight increase in swelling, facilitating the diffusion of the dye into the hydrogel interior. As a result, the amount of DB78 adsorbed at equilibrium increases.

The results obtained suggest that the highest DB78 retention capacity in wastewater is achieved with cellulose-based hydrogels prepared with ECH at 125% (relative to cellulose), a cross-linking time of 4 h, and an estimated temperature of 80 °C. For IPN-type hydrogels, the optimum should be recorded for those containing 90% cellulose. To confirm these hypotheses, further studies will be conducted, with the main objective of examining the behavior of these hydrogels under various usage conditions, including their ability to retain other dyes from industrial wastewater.

**Figure 8:**
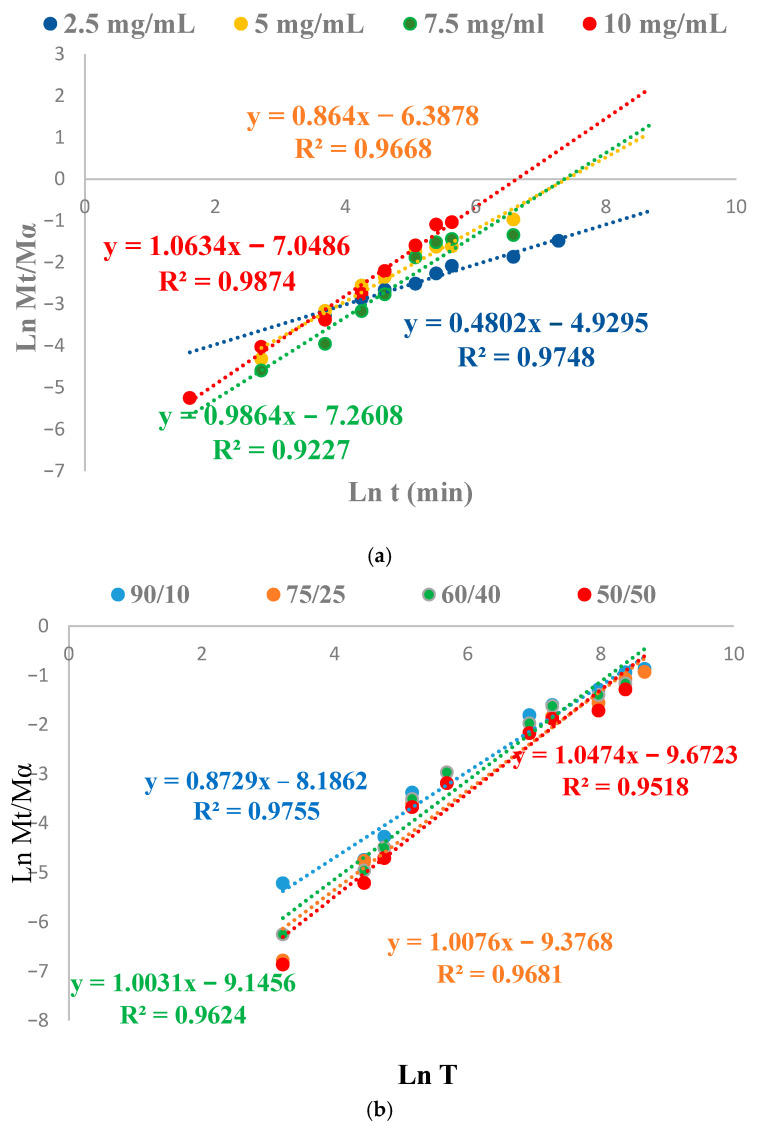
Application of the Peppas model to determine the transport mechanism depending on the initial dye concentration (**a**) and PN hydrogel composition (**b**).

In cellulose-based hydrogels, the rate of water diffusion tends to decrease with increased cross-linking density and reduced porosity. Figure 8 presents the Peppas model analysis used to evaluate the transport mechanism, considering different initial dye concentrations for adsorption onto cellulose-based hydrogels and onto hydrogels prepared from various compositions of the interpenetrating polymer network (IPN).

The adsorption kinetics were analyzed using the Ritger–Peppas model, which elucidates different transport mechanisms depending on the situation, the hydrogel composition, and the initial dye concentration. For cellulose-based hydrogels, the diffusion exponent n = 0.5 indicates a predominantly Fickian diffusion transport mechanism at an initial dye concentration of 2.5 mg/mL, consistent with the higher cross-linking density and lower porosity of the network, which slows the diffusion of water and the solute. As the dye concentration increases, the exponent n approaches 0.86 at 5 mg/mL, suggesting a non-Fickian transport mechanism driven by both diffusion and polymer network relaxation. For higher dye concentrations, of 7.5 mg/mL and 10 mg/mL, respectively, the values of n ≈ 1 indicate a Case II transport mechanism, dominated by swelling and relaxation processes of the hydrogel network. For the cellulose–PVA IPN hydrogels, the n exponent values are also close to 1, indicating a non-Fickian (Case II) transport mechanism, dominated by swelling and relaxation processes of the polymer network. This interpretation is further supported by the increased porosity of the composite hydrogels, which enables the swift ingress of water, as well as by the nearly linear correlation observed between qe (mg/g hydrogel) and Ce (mg/L), indicating a volumetric distribution of the dye within the hydrogel matrix. These combined kinetic and structural considerations reinforce the conclusion that diffusion is a major factor in the adsorption of DB78, but the hydrogel’s structure and architecture strongly influence the primary transport mechanism.

To determine whether the adsorption process is better described by Langmuir or Freundlich behavior, the experimental adsorption data were analyzed using both isotherm models, as commonly reported in the literature [68].

Freundlich model:

(7) $$q_{e}=K_{F}\times C_{e}^{1/n}$$

$q_{e}$—amount of dye adsorbed at equilibrium (mg/g);

Ce—concentration of the dye in the solution at equilibrium (mg/L);

$K_{F}$—Freundlich constant (adsorption capacity);

1/n—empirical exponent indicating the intensity/favorability of adsorption.

The data used to calculate the Freundlich model are shown in Table S3 (from the Supplementary Material), and the graphical representation of the Freundlich model (Figure S1). The Freundlich model parameters obtained from the experimental data are 1/n = 1.0365 and Kf approximately 0.0326. These parameters offer insights into the adsorption characteristics of the dye–hydrogel system. The value of 1/n, which indicates adsorption intensity, is close to unity and slightly exceeds 1, suggesting a deviation from the ideal adsorption behavior predicted by the Freundlich model and potentially indicating non-ideal adsorption phenomena, possibly influenced by interactions between adsorbed dye molecules or alterations in surface affinity at higher concentrations. The Freundlich constant Kf, which is related to adsorption capacity, is relatively low, indicating that the hydrogel has limited adsorption capacity for this dye under the specified experimental conditions. For clarity, the Freundlich adsorption model was also presented in a linearized correlation form by plotting q_e_ versus C_e_, as shown in Figure S2 (Supplementary Material).

The relationship between the equilibrium adsorption capacity (q_e_ mg/g of hydrogel) and the equilibrium concentration (C_e_ mg/L) is nearly linear within the investigated concentration range (Figure S1—Supplementary Material), with a coefficient of determination R^2^ = 0.906. This trend indicates a dye distribution mechanism within the hydrogel network rather than monolayer adsorption at specific binding sites. Such behavior is characteristic of swollen hydrogels, where dye solution uptake is controlled by diffusion and polymer network relaxation. These observations are consistent with the kinetic analysis performed using the Peppas model for adsorption, which indicates a dye transport dominated by network relaxation and swelling (n is 1 or very close to this value), especially for samples containing PVA and for those where the dye solution concentration was 7.5 mg/mL and 10 mg/mL.

The adsorption process is not adequately described by either the Langmuir model or the classic Freundlich model. The experimental data do not show a clear saturation region, as required by the Langmuir model, and the relationship between q_e_ and C_e_ is nearly linear over the investigated concentration range. This behavior indicates a dye-distribution mechanism within the hydrogel network, characteristic of swollen hydrogels, in which dye uptake is controlled by diffusion and polymer network relaxation rather than monolayer adsorption on predefined interaction zones. The Freundlich model was used only as a complementary analysis; the parameters obtained suggest behavior close to linearity, but their interpretation should be approached with caution, given the limited number of points and the concentration range analyzed. Unlike the systems discussed in the reference literature [68], where equilibrium data are available over a wide concentration domain (C0), and adsorption saturation is observed, our system is dominated by matrix relaxation mechanisms, swelling, and the dye’s volumetric distribution.

### 3.5. Performance of Reported Hydrogels Compared to Other Materials

As is known, the dyes in the Direct Blue class (in our case, DB78) are direct anionic dyes. The materials that give the best retention are those based on cellulose in combination with other cationic polymers (chitosan, polyaniline, etc.) or on carbon/TiO_2_ composites, which rely on electrostatic attraction, π-π bonds, etc. As for cellulose-based hydrogels, few studies have reported on the adsorption of the DB78-type dye. However, the efficiency of dye removal is generally low (10–30%), with equilibrium reached after 72 h. In the study by Shoaib et al., a cellulose-based hydrogel was obtained by simple gelation of the polysaccharide dissolved in a LiCl/dimethylacetamide mixture at 80 °C for 24 h, followed by freeze-drying. This hydrogel can retain up to 53.76 mg of a DB class dye (specifically DB86) per gram, achieving an adsorption efficiency of up to 30%. Adsorption efficiency depends on factors such as the initial dye concentration, pH, and the amount of adsorbent used [69].

The use of other polysaccharides, alone or in combination with cellulose, results in materials with sufficiently high adsorption capacity. Murcia-Salvador et al. use as adsorbents chitosan spheres obtained by gelling a chitosan solution in acetic acid, when dropped into a NaOH solution, and a β-cyclodextrin polymer obtained in the presence of epichlorohydrin [43]. The maximum adsorption capacity of DB 78 was found to be 12.3 mg/g for the chitosan-based adsorbent, respectively 23.47 mg/g for the cyclodextrin-based polymer, depending on the initial concentration of the dye solution. Chitosan powder, chitosan spheres mixed with poly(acrylamide), and adsorbed DB78 were used, and the average efficiency was determined; the synthetic polymer in the combination increased chitosan’s capacity to adsorb the dye [42]. The studies demonstrated approximately equal dye adsorption efficiency (94.4% and 94.1%, respectively), but the powdered polysaccharide had a retention capacity of 10.5 mg/g, compared to 26.3 mg/g for its combination with the synthetic polymer. The authors concluded that the polyacrylamide gel enhances chitosan’s adsorption capacity.

Sponges based on chitosan and ZnO, deposited on a polysaccharide via Atomic Layer Deposition, were obtained by Gubitosa et al. [70]. They showed a very high retention capacity of DB 78 from aqueous solutions. The influence of factors such as dye concentration, pH, ionic strength, and temperature was systematically investigated. Increasing the adsorbent amount and the dye concentration favors its removal from water. The maximum adsorption capacity was 2000 + 400 mg/g, which was obviously favored by the adsorbent’s cationic sites. It should be noted that the same performance was recorded in the removal of mixtures of azo dyes, namely Direct Red 83:1 and Direct Yellow 86.

The little information available in the literature regarding the retention capacity of anionic dyes, especially DB78, by cellulosic materials, but also those regarding the adsorbent quality of other polysaccharide-based materials, which entitles us to consider that the hydrogels we report in this paper have an average, but promising performance in the use as adsorbents for the purification of waters from the textile industry, polluted with direct anionic dyes. It should be noted that these materials are derived from inexpensive, commercial polymers, one of which (cellulose) is derived from renewable sources.

## 4. Conclusions

In this study, cellulose-based hydrogels (HC) and Cel/PVA interpenetrating polymer networks (IPNs) were successfully synthesized using epichlorohydrin (ECH) as a cross-linking agent. The results showed that synthesis parameters, particularly ECH ratio, temperature, and cross-linking duration, significantly affected the materials’ swelling behavior and structural properties. Optimal ECH content enhanced hydrophilicity and swelling capacity, while PVA incorporation improved the porosity of the IPN network. The IPNs exhibited high adsorption efficiency for DB78 dye, highlighting the role of cellulose’s affinity and the effect of PVA. These findings demonstrate the potential of cellulose and, especially, Cel/PVA IPNs as sustainable and effective materials for dye removal in wastewater treatment.

## Figures and Tables

**Table 1:** Experimental plan for cellulose hydrogels obtaining.

Sample Codes *	Synthesis Parameters *		
	ECH (%, *w*/*w* of Cellulose)	Temperature (°C)	Duration (h)
HC1	50	60	5
HC2	75		
HC3	100		
HC4	125		
HC5	75	50	6
HC6		60	
HC7		70	
HC8		80	
HC9		70	4
HC10			5
HC11			7

* Concentration of cellulose solution: 3% (*w*/*w*).

**Table 2:** The n, k, and τ values were determined by integrating the swelling data from different kinetic diffusion models.

Sample	Qmax	Peppas Model			First-Order Kinetics		Second-Order Kinetic		Voight Equation	
		n	k	R^2^	K (h)	R^2^	k	R^2^	τ (h)	R^2^
HC3	80.80 ± 0.7	0.8	0.64	0.8204	1.009	0.8053	4.72	0.9985	0.76	0.9538
HC4	70.73 ± 0.5	0.6033	0.4524	0.9717	0.59	0.9787	1.386	0.9974	1.71	0.8799
HC2	63.33 ± 0.21	0.5313	0.4515	0.9795	0.4682	0.979	1.37	0.9991	1.79	0.9566
HC1	59.33 ± 0.21	0.5869	0.447	0.9371	0.5129	0.959	1.36	0.9753	1.78	0.9206
Cel/PVA = 60/40	213.5 ± 1.67	0.136	0.83	0.9202	0.87	0.9344	2.62	0.9998	0.19	0.9478
Cel/PVA = 75/25	188.03 ± 1.46	0.1	0.8	0.9316	0.38	0.958	1.96	0.9998	0.2	0.917
Cel/PVA = 90/10	135.13 ± 1.79	0.144	0.77	0.86	0.3016	0.8399	1.92	0.9998	0.49	0.9338

## Data Availability

The data presented in this study are available on request from the corresponding author.

## Supplementary Materials

The following supporting information can be downloaded at: https://www.mdpi.com/article/10.3390/polym18030346/s1, Figure S1: Graphical representation of the Freundlich model; Figure S2: Linear correlation between q_e_ and C_e_ for the Freundlich adsorption model, q_e_ = 0.042C_e_ + 5.649; Table S1: The factors that influenced the swelling degree value, Q%; Table S2: Average values of DB78 adsorption efficiency depending on the value of the hydrogel synthesis parameter; Table S3: Adsorption data used to calculate the parameters of the Freundlich and Langmuir models.
