# Supplementary material for: Synthesis and Characterization of Cellulose and IPN (Cellulose/PVA) Hydrogels and Their Application in Dye Retention
Source: Polymers (Basel). 2026 Jan 28;18(3):346. doi: 10.3390/polym18030346 (PMC12899320; doi:10.3390/polym18030346)
Supplement: Supplementary file 1 [file polymers-18-00346-s001.zip › Supplementary material.pdf]

Supplementary material

The article's title**: Synthesis and characterization of Cellulose and IPN (cellulose/ PVA) hydrogels and their application in dye retention**

Authors: **Meriem Mihoub, Salah Hamri, Marcel Popa, Camelia Elena Tincu (Iurciuc), Tewfik Bouchaour, Lamia Bedjaoui-Alachaher, Usman Abubakar Katsina, Mutawakkil Muhammad**

Table S1. The factors that influenced the swelling degree value, Q%

**Factors influencing the swelling degree value, Q%**				
**Cross-linking agent concentration, %**	**100%**	**125%**	**75%**	**50%**
Average value of the maximum swelling degree value, Q%	80.8±0.7	70.7±0.5	63.3±0.21	59.33±0.21
**Temperature, ^{0}C**	**80^{0}C**	**70^{0}C**	**60^{0}C**	**50^{0}C**
Average value of the maximum swelling degree value, Q%	81.96±0.42	79.4±0.36	77.43±0.35	75.43±0.35
**Cross-linking time, h**	**4h**	**5h**	**7h**	
Average value of the maximum swelling degree value, Q%	99.13±0.32	95.13±0.32	85.13±0.32	
**Weight ratios Cel/PVA (w/w)**	**60/40**	**75/25**	**90/10**	
Average value of the maximum swelling degree value, Q%	213.5±1.67	188.03±1.45	135.13±1.79	

Table S2. Average values of DB78 adsorption efficiency depending on the value of the hydrogel synthesis parameter

Parameter	Value	Maximum (average) amount of DB78 adsorbed (mg)	Mass of hydrogel used (g)	Maximum (average) amount of DB78 adsorbed/mass of hydrogel (mg/g)	Maximum (average) adsorption efficiency
Cel/PVA ratio (w/w)	90/10	10.5	0.145	72.3	42%
	75/5	9.87	0.142	69.4	39.50%
	60/40	8.8	0.181	48.46	35.20%
	50/50	7.13	0.157	45.5	28.50%
Cross-linker (ECH) concentration (% of cellulose)	125	9.05	0.237	38.1	36.20%
	100	6.125	0.217	28.2	24.50%
	75	3.275	0.273	12	13.10%
	50	1.875	0.213	8.8	7.50%
Temperature (^{0}C)	50	13.25	0.109	121.5	53%
	60	14.6	0.104	140.2	58.40%
TBG solution concentration (mg/ml)	2.5	8	0.232	34.4	32%
	5	10.5	0.145	72.3	42%
	7.5	15.75	0.098	160.5	45%
	10	28	0.13	215.2	56%

Table S3. Adsorption data used to calculate the parameters of the Freundlich and Langmuir models

C0 mg/mL	C0 mg/L	Hydrogel weight (g)	qe mg/g	Average Ce mg/ml	Average Ce mg/L	Ce/qe	ln qe	ln Ce
2.5	2500	0.232	34.4	0.9	900	26.16	3.53806	6.802
5	5000	0.145	103.4	2.0014	2001.4	19.36	4.6386	7.602
7.5	7500	0.098	160	4.364	4364	27.28	5.07517	8.381
10	10000	0.13	215	4.41	4410	20.51	5.37064	8.392

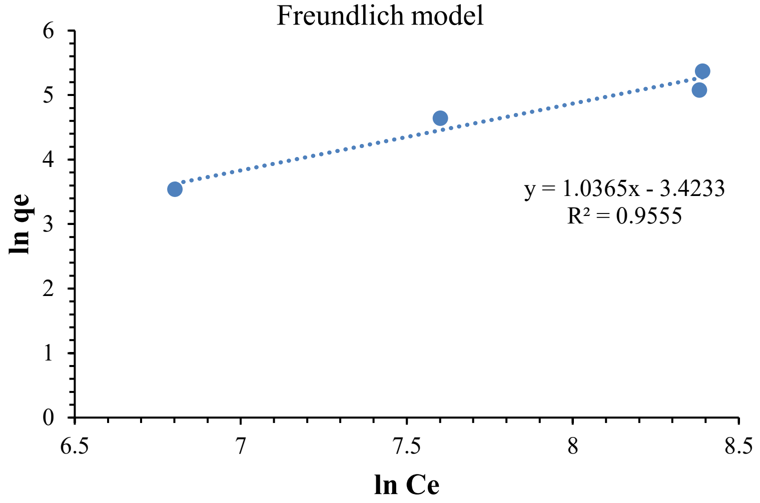

Figure S1. Graphical representation of the Freundlich model

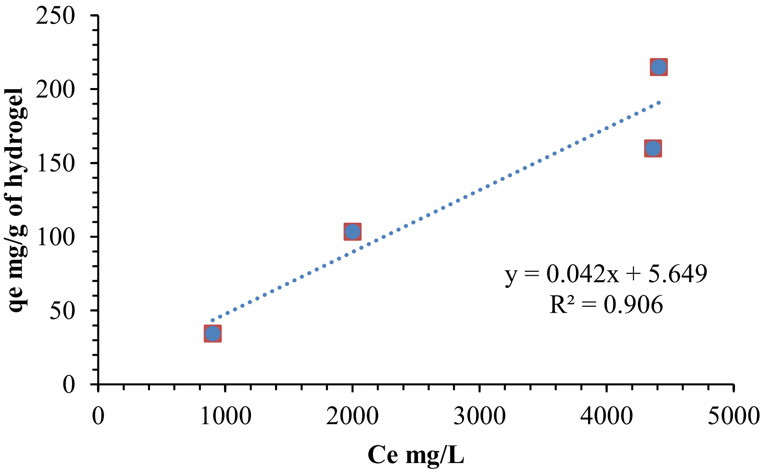

Figure S2. Linear correlation between q_e and C_e for the Freundlich adsorption model, $q_e = 0.042C_e + 5.649$
